# Demographic, Clinical, and Serological Characteristics of Antiphospholipid Syndrome Patients From the Anticoagulation Clinic of Hospital Universitario San Vicente Fundación, Medellín, Colombia

**DOI:** 10.7759/cureus.35114

**Published:** 2023-02-17

**Authors:** Santiago Álvarez-López, María Fernanda Ariza-Gómez, Vanessa López-Montoya, John Ubeimar Cataño-Bedoya, Diana Giraldo-Mendez, Fabian Jaimes

**Affiliations:** 1 Department of Internal Medicine, University of Antioquia, Medellín, COL; 2 Department of Vascular Specialist, University of Antioquia, Medellín, COL; 3 Department of Vascular Specialist, CES Clinic, Medellín, COL

**Keywords:** antiphospholipid antibodies, lupus anticoagulant, antibodies anticardiolipin, venous thrombosis, antiphospholipid syndrome

## Abstract

Introduction: Antiphospholipid syndrome (APS) is an acquired autoimmune thrombophilia, characterized by vascular thrombosis or obstetric compromise, associated with the presence of antiphospholipid antibodies. Large international studies have analyzed the clinical/serological behavior of the disease and in Colombia, there are few cohorts that have been evaluated.

Objective: The main objective is to characterize the patients with APS followed in the anticoagulation clinic of a tertiary care hospital and to determine the clinical manifestations and serological findings at diagnosis.

Materials and methods: A retrospective descriptive study was carried out to evaluate patients with a presumptive and/or confirmed diagnosis of APS, according to modified Sapporo criteria, which fulfilled the inclusion and exclusion criteria established by the authors. The information was collected from the review of medical records.

Results: We included 103 patients, with the female sex being the most prevalent (86.6%). 54.3% of the patients (n = 56) had a diagnosis of primary APS. Venous thrombotic events occurred in 87.3% (n = 90) of the patients, 34.9% (n = 36) had arterial thrombosis (n = 36), and 3.9% (n = 4) had catastrophic APS (n = 4). 15 cases of Obstetric APS were documented. Lupus coagulation inhibitor (LA) positivity was the most prevalent marker in 84% (n = 68) of cases.

Conclusions: The clinical behavior in this cohort of patients is like that found in large international and national studies. Most patients have a probable diagnosis of APS, so they could overestimate the real prevalence and condition of long-term anticoagulant treatment.

## Introduction

Background

Antiphospholipid syndrome (APS) is an acquired autoimmune thrombophilia, distinguished by thrombosis (venous, arterial, and microvascular) and/or obstetric morbidity [1]. As is known, APS has a four times higher prevalence in women, and it is an entity suspected in young patients with thrombosis, such as patients younger than 50 years with stroke - who up to 20% may be diagnosed with APS [2]. This syndrome may be associated with other autoimmune diseases such as systemic lupus erythematosus (SLE), infections, drugs, and malignancy, or it may present as a primary disorder [3]. Usually, diagnosis depends on serological tests such as lupus anticoagulant (LA), anti-cardiolipin antibodies (aCL), or anti-B2 glycoprotein-I antibodies (anti-B2GPI) [3].

In 1999, Sapporo clinical and laboratory criteria for APS classification were created [4], which were updated in 2006 when certain clinical aspects (livedo reticularis, thrombocytopenia, nephropathy, valvular cardiac disease, hemolytic anemia, etc.) could be “extra” criteria [5]. Current criteria for APS diagnosis include two clinical criteria (venous/arterial thrombosis in absence of vasculitis, and pregnancy-related complications) and three serological markers (LA, IgG/IgM aCL, IgG/IgM anti-b2GPI) [6]. From a consensus standpoint, an APS diagnosis is acknowledged when at least one of the two clinical criteria and one of the three laboratory criteria are present [6]. Nonetheless, APS may be variable among patients, i.e., triple-positive patients may present with recurrent thrombosis in up to 30% of cases [2]. This clinical and laboratory spectrum may lead to delays in diagnosis and serious implications for the patient, as classification is essential to choose the most appropriate therapeutic strategy [7,8]. 

The best study available to understand the demographical, clinical, and serological variables for APS diagnosis is the Euro-Phospholipid Project [9], with a population obtained from 13 European countries, which makes the results questionable for application in Latin America [8]. Moreover, little data from Latin America about this condition is available; even though multicentric studies have been performed, a small sample size remains an issue [8,10,11].

Furthermore, the anticoagulation clinic from the Hospital San Vicente Fundación (HUSVF) was created in 1975 and currently provides services for 450 patients with different diseases. It’s the largest clinic in the country and serves as a reference center for neighboring departments. Approximately 15% of patients have received or currently receive anticoagulation as APS treatment [12,13].

Objectives

The present study wanted to characterize the APS patients from the anticoagulation clinic of the HUSVF, with the objective of comparing the results with the European study and producing foundations for further studies.

## Materials and methods

Study design

This is a retrospective study, based on a cohort of patients with APS diagnosis from the anticoagulation clinic at the HUSVF. Patients treated from January 2013 to December 2018 were included. As long as there was sufficient information, the patient was included in the analysis. Data collection was performed by researchers. The present study was performed after ethics committee approval; no informed consent was taken due to its retrospective nature.

Population

All patients with APS diagnosis (primary or secondary), older than 18 years, who attended clinical follow-up at the anticoagulation clinic and had at least two appointments between January 2013 and December 2018, were included. Patients without sufficient data for classification, those with telephonic follow-up, or comorbidities which prohibited anticoagulation therapy, were excluded. The data source was clinical charts. Patient selection underwent several filters: 1) we made sure that each patient had an APS diagnosis (Table 1) [3] as the follow-up cause; 2) data for APS diagnosis was available for each patient on clinical charts. If these two conditions were met, the patient was included in the database. Follow-up frequency was selected by Vascular specialists in charge of the anticoagulation clinic.

**Table 1:** Classification criteria for antiphospholipid syndrome. aCL: anticardiolipin antibodies; Anti-b2GPI: anti B2 glicoprotein antibodies; GPL or MPL: units for IgG or IgM antibodies [3]

Vascular thrombosis	One or more clinical episodes of arterial, venous or small vessel thrombosis in any tissue or organ, objectively validated.
Pregnancy morbidity	One or more unexplained death in morphologically normal fetus at 10 weeks of gestation or more.
	One or more premature birth of morphologically normal neonates at 34 weeks of gestation or earlier, due to eclampsia, severe pre-eclampsia or severe placental insufficiency.
	Three or more unexplained consecutive spontaneous abortions before the 10th week of gestation, excluding anatomic maternal/hormonal abnormalities, and excluding chromosomic abnormalities in both parents.
Laboratory criteria	Presence of antiphospholipid antibodies, in two or more occasions, at least 12 weeks apart and no more than 5 years before clinical presentation, proven by one of the following:
	Lupus anticoagulant
	aCL of IgG and or IgM isotype in serum or plasma present in medium or high titer (> 40 GPL or MPL, or > 99th percentile).
	Anti-b2GPI of IgG isotype in serum or plasma > 99th percentile.

Variables

Socio-demographical, clinical, and laboratory variables were included in the present study. Data were extracted from clinical charts from the anticoagulation clinic, or clinical charts from in-patient stances of each patient. Clinical variables were divided into venous/arterial thrombosis, pregnancy morbidity, and severe complications of the disease (catastrophic APS). Serological variables included diagnostic markers (LA, aCL, anti-b2GPI), platelet count at diagnosis, coagulation tests (prothrombin time, partial thromboplastin time, INR), and factor II activity in case it was available. Outcomes were the frequency and distribution of each variable previously mentioned. Screening LA was considered positive for patients with a value higher than 1.2. Moreover, confirmatory LA was classified as weak (values between 1.2 and 1.5), moderate (1.5-2.0), and strong (> 2.0). These categories were used to interpret both screening and confirmatory LA distribution.

Source and data measurement

All data were extracted from clinical charts, either from the anticoagulation clinic or the in-patient stances of the patient. The collection was performed through a pre-designed Microsoft Office Excel template. The data registry was performed through pre-selected ranges to avoid typing errors. Absolute and relative frequencies were calculated for categorical variables. Quantitative variables are presented as median with interquartile ranges.

Sample and sample size

Given the low frequency of the disease, no formal sample size was calculated. However, based on the descriptive interest of our study, various practical considerations were made to the total population of the anticoagulation clinic from January 2013 to December 2018 was 1,298 patients. 123 patients were followed up due to an APS diagnosis. Finally, 103 met the criteria to be included in the study.

Bias

As this is a retrospective study, data availability was limited to what was readily available in clinical charts. Given the study population, the distribution and behavior of obstetric APS may be under-registered; hence, no conclusions may be drawn regarding isolated obstetric APS. In addition, the extraction and consignment of data could be altered by human error.

## Results

Population

**Figure 1:**
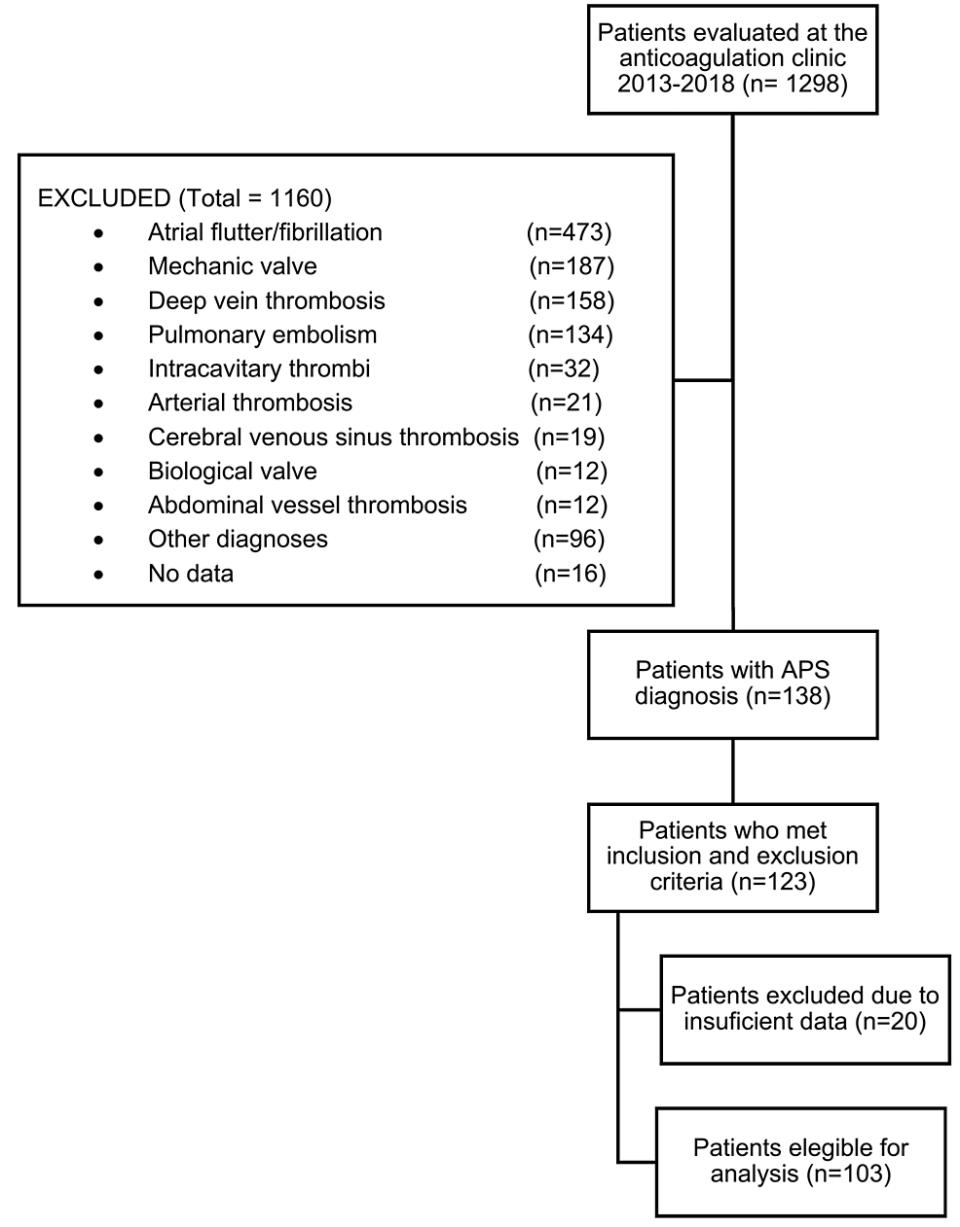
Participant selection flowchart

During the period between January 2013 and December 2018, 2,311 visits to the anticoagulation clinic were registered, corresponding to 1,298 patients. Follow-up ICD-10 diagnoses were filtered through the hospital management system software, and 138 with APS diagnoses were found. After evaluating inclusion and exclusion criteria, 123 were eligible. After evaluating all data available (patients in whom APS was ruled out), a definitive sample of 103 patients was obtained (Figure 1).

Descriptive Data

83.3% (n = 86) of patients were female, with an age between 20 and 39 years in 44.6% (n = 46) of patients at the moment of APS diagnosis. The place of provenance is unknown (urban or rural) in more than 80% of the included sample. Only 35.9% (n=37) of patients continue under follow-up at the anticoagulation clinic (Table 2).

**Table 2:** Clinical and demographical variables upon APS diagnosis LA: lupic anticoagulant; aCL: anticardiolipine antibodies; ANCA: antineutrophil cytoplasmic antibodies.

Characteristics (n= 103)	Frequency	
Age	n	%
Median (years)	37,5	
Femenine - No. (%)	86	83,4
Primary APS No. (%)	56	54,3
Secondary APS		
Associated disease		
Systemic lupus erythematosus	41	87
Rheumatoid arthritis	2	4,3
Mixed connective tissue disease	2	2,2
Vasculitis **	2	2,2
Venous thrombosis No. (%)	90	87,3
Lower limbs	53	74,6
Pulmonary embolism	19	34,5
Upper limbs	8	11,2
Cerebral venous sinus thrombosis	5	7
Other	5	7
Arterial thrombosis No. (%)	36	34,9
Stroke	25	45,4
Lower limbs	7	12,7
Acute myocardial infarction	2	3,6
Other	2	7
Obstetric morbidity No. (%)	15	14,5
One or more abortions >10 weeks	6	40
Three or more abortions < 10 weeks	3	20
Fetal demise > 20 weeks	1	6,6
Pregnancy related hypertensive disorders	1	6,6
Several of the previous	4	26,6
Serological variables		
Screening LA	81	78,6
Positive (>1,2)	68	84
Confirmatory LA	64	94,1
Weak (1,2- 1,5)	12	16,2
Strong (>2,0)	27	36,4
Moderate (1,5 - 2,0)	25	33,7
ACL IgM	90	87,4
Positive	38	42,3
ACL IgG	90	87,4
Positive	46	51,1
Anti B2 glicoprotein-I IgM	28	27,2
Positive	12	42,9
Anti B2 glicoprotein-I IgG	42	40,1
Positive	20	47,6
Triple positive markers	5	4,9
Rethrombosis	15	14,5
Unknown	6	54
Suspension	5	34
Change to rivaroxaban	1	6,6
Other causes	3	19,9
** Includes ANCA associated vasculitis and cryoglobulinemic vasculitis		

Outcomes

Clinical Criteria

45.6% (n=47) of patients had a diagnosis of secondary APS, being SLE the most commonly associated disease (87.2%, n=41). Out of all clinical events which led to APS diagnosis, venous thromboembolism was the most common one, up to 87.3% (n=90) of the population. 34.9% of patients presented with arterial thrombosis (n=36), being stroke the most common form in up to 45.4% (n=25). 3.9% of patients presented catastrophic APS (n=4) (Table 2). 

Obstetric APS

Fifteen patients (14.5%) presented with obstetric events, explained primarily by one or more abortions at the tenth week or more of gestation (40%, n=6). Given that obstetric manifestations did not present as an isolated entity, Table 3 distinguishes the associated thrombotic events.

**Table 3:** Thrombotic events associated with obstetric APS

Thrombosis type	n	%
Venous	14	93.3
Arterial	9	60
Venous and arterial	8	53,3
Re-thrombosis	2	13,3
Catastrophic APS	2	13,3

Serological criteria

Screening LA was the most prevailing finding, in 84% of patients (n=68), with a positive confirmatory LA in 94.1% of cases (n=64). ACL was positive for the IgG isotype in 51.5% of cases (n=46), whereas the IgM isotype was positive in 42.3% of patients (n=38). Unfortunately, over 50% of patients had no registry of IgG/IgM antiB2GPI. Triple positivity was detected in five patients (4.9%); however, clinical characterization was possible only in four of them (Table 4).

**Table 4:** APS patients with triple-positive serological markers (*) TMA: thrombotic microangiopathy; (**) TR: therapeutic range for warfarin

	Patient 1	Patient 2	Patient 3	Patient 4
Gender	Feminine	Feminine	Feminine	Feminine
Type of APS	Primary	Primary	Secondary to SLE	Primary
Venous thrombosis	Lower limbs DVT	Bilateral DVT	Lower limbs DVT and suprahepatic veins thrombosis	Lower limbs DVT
Arterial thrombosis	PE of sub segmental and segmental arteries	PE of sub segmental arteries	No	No
Obstetric morbidity	Seven abortions < 10 weeks gestation and one abortion > 10 weeks gestation	Five recurrent abortions < 10 weeks gestation	No	No
Re-thrombosis	No	PE while on warfarin (TR)**	Portal thrombosis while on warfarin (TR)**	No
Catastrophic APS	Renal TMA* and acute coronary syndrome	Renal TMA* and central retinal vein occlusion	No	No
Outcome	Alive, anticoagulated with warfarin	Alive, anticoagulated with warfarin	Alive, anticoagulated with rivaroxaban	Alive, anticoagulated with warfarin

Non-criteria manifestations

28% of patients presented with thrombocytopenia (n=29), being moderate thrombocytopenia (platelet count between 50.000 and 100.000) the most common one (57%, n=15). Only in 17 clinical charts, other clinical manifestations were described, such as lower limbs ulcers and livedo reticularis in 3.9% of patients (n = 4 for each event).

INR follow-up and re-thrombosis

During the first year of follow-up, only 52.4% of patients with APS diagnosis (n=54) had INR in the therapeutic range (between 2 and 3). Re-thrombosis occurred in 14.5% of patients (n=15). In most cases, no cause was determined. Other causes of re-thrombosis are shown in Table 5.

**Table 5:** Causes of re-thrombosis in patients with APS evaluated at the HUSVF * Switch to rivaroxaban

Cause	No.	(%)
Unknown	6	54
Warfarin suspension	5	34
Other causes	2	13,3
Switch to direct anticoagulant *	1	6,6
Permanence out of therapeutic range	1	6,6

Evaluation of factor II activity

Thirteen patients were followed up with at least one measurement of factor II activity, due to a labile INR or due to non-credible coagulation tests. Of such patients, 84.6% (n=11) were in the therapeutic range or over-anticoagulated. Despite this, 30.7% (n=4) presented with re-thrombosis during follow-up. Nonetheless, only 23% (n=3) had a strict follow-up with factor II activity and none of these patients presented re-thrombosis.

## Discussion

The present study describes clinical and serological characteristics which led to APS diagnosis in 103 patients evaluated from 2013 to 2018 at the anticoagulation clinic from the HUSVF. A higher prevalence of APS was observed in female patients (83%), and the most common thrombotic event was lower limb DVT (51.4%). Regarding serological markers, 51% of patients had a positive IgG aCL.

Three internationally relevant studies are currently available: the Euro-Phospholipid Project (n=1,000) [9], the one published by the Korean health agency HIRA (n=3,088) [14], and for Latin America the multicentric work by Mejía-Romero et al. (n=100) [12]. Two Colombian studies are available: one by Grajales-Ramirez et al., published in 2004 [15], and Cañas-Osio et al., in 2010 [16], both of which describe clinical and serological characteristics of 32/62 APS patients, respectively. Re-thrombosis was not evaluated, nor was the anticoagulation therapy for such patients. 

These studies, just like our study, have described a higher frequency of APS in female patients in the fourth decade of life, with an equivalent distribution for primary/secondary APS. DVT was present in 50% of our patients, a higher frequency than in the European (31%) [9] and Korean (20%) [14] studies. Similarly, arterial thrombotic events (mainly stroke) were more frequent in our cohort (45%) compared to 13.1% and 27.3% for the European [9] and Korean [14] studies, respectively. This finding could suggest a higher predisposition for thrombotic events in our patients, although this should be confirmed with further studies.

Current guidelines suggest IgG/IgM antiB2GP-I and aCL as serological markers, however, the relevance of IgM isotypes of these antibodies has been debated [17]. A systematic review by Devresse et al. [17] found a significant association between thrombotic complications (both arterial and venous) and IgG positivity. This was replicated in our cohort, where 100% of patients with arterial thrombotic events were positive for IgG aCL. Unfortunately, not enough data was available to determine the prevalence of positive anti-B2GP-I and associated thrombotic events.

Obstetric APS did not present as an isolated entity in our study, as 66% of patients had a previous history of thrombotic and/or arterial events, even though these characteristics were not evaluated on international cohorts. In 2010, Bramham et al. [18] published a cohort of 67 obstetric APS patients, stratified by obstetric outcomes and the presence of thrombotic events. In the group with thrombotic events, worse outcomes were observed, including catastrophic APS, with a statistically significant correlation. Latino et al. [19] concluded that these scenarios (thrombotic APS and obstetric APS) were both characterized by the presence of triple-positive antiphospholipid antibodies. These findings are consistent with the present study, where 100% of patients who had both obstetric and thrombotic APS had triple-positive antiphospholipid antibodies.

Regarding the recurrence of thrombotic events in APS patients, the risk seems to be associated with the involved vascular bed and the antiphospholipid antibodies profile [20]. Pengo et al. [21] retrospectively analyzed 160 APS patients with triple-positive antibodies, to determine profiles with a higher risk for re-thrombosis. No factor was associated with such adverse outcome (44% at 10 years follow-up) [21]. These findings were replicated in our study, where 54% of patients had re-thrombosis, although no cause was identified.

Finally, follow-up based on factor II activity is recommended in patients who achieve a stable INR [22,23]. Appropriate anticoagulation is considered when factor II activity is between 15% and 25%. Nonetheless, this assay is not widely available; it has been used in those patients who could not achieve a therapeutic INR or those with a labile INR. In the present study, a higher rate of re-thrombosis was observed in those patients followed up with factor II activity (30.7% vs 12.4%). This could be due to less time in the therapeutic INR range, as the patients with strict follow-up based on factor II activity did not present such adverse outcomes. Data quality was poor, which could be corrected by a prospective study in this scenario.

Shortcomings

Given the retrospective nature of our study, the main limitation was the incomplete clinical and serological data, due to incomplete clinical charts, extra-institutional diagnosis, or old data. In our case, the main issue was an incomplete registry of APS serological markers, which prevented a complete analysis of each of the variables. As our patients were evaluated in an anticoagulation clinic, we suppose that adverse obstetric events do not represent the actual behavior in the population, compared to high-risk obstetric clinics.

## Conclusions

APS is a disease with a high morbidity burden. The socio-economic condition of the patients, as well as comorbidities, pose a limitation for correct disease follow-up and may need certain measures for underprivileged populations who require medical attention. The demographic and clinical characteristics of our patients are similar to those reported in larger international cohorts. Nonetheless, a higher prevalence of venous/arterial thrombotic events was observed. Moreover, the recurrence risk of thrombosis in these patients is not negligible, even when the correct therapy is received.

Although certain serological markers seem to confer a higher risk for complications, the difficulties in data collection led to doubtful conclusions. A complete serological panel should be performed in the diagnostic process, as the lack of a single one may be the difference between proper treatment and adverse outcomes. Follow-up of patients who may not achieve INR goals may be ineffective if performed through factor II activity, hence, trustworthy tools for this task should be pursued.
